# Myeloid-derived suppressor cells impair CD4+ T cell responses during chronic *Staphylococcus aureus* infection via lactate metabolism

**DOI:** 10.1007/s00018-023-04875-9

**Published:** 2023-07-22

**Authors:** Oliver Goldmann, Eva Medina

**Affiliations:** Infection Immunology Research Group, Helmholtz Centre for Infection Research, 38124 Braunschweig, Germany

**Keywords:** *Staphylococcus aureus*, Myeloid-derived suppressor cells (MDSC), CD4+ T cells, Lactate, NAD+/NADH redox, SCENITH

## Abstract

*Staphylococcus aureus* is an important cause of chronic infections resulting from the failure of the host to eliminate the pathogen. Effective *S. aureus* clearance requires CD4+ T cell-mediated immunity. We previously showed that myeloid-derived suppressor cells (MDSC) expand during staphylococcal infections and support infection chronicity by inhibiting CD4+ T cell responses. The aim of this study was to elucidate the mechanisms underlying the suppressive effect exerted by MDSC on CD4+ T cells during chronic *S. aureus* infection*.* It is well known that activated CD4+ T cells undergo metabolic reprogramming from oxidative metabolism to aerobic glycolysis to meet their increased bioenergetic requirements. In this process, pyruvate is largely transformed into lactate by lactate dehydrogenase with the concomitant regeneration of NAD+, which is necessary for continued glycolysis. The by-product lactate needs to be excreted to maintain the glycolytic flux. Using SCENITH (single-cell energetic metabolism by profiling translation inhibition), we demonstrated here that MDSC inhibit CD4+ T cell responses by interfering with their metabolic activity. MDSC are highly glycolytic and excrete large amount of lactate in the local environment that alters the transmembrane concentration gradient and prevent removal of lactate by activated CD4+ T. Accumulation of endogenous lactate impedes the regeneration of NAD+, inhibit NAD-dependent glycolytic enzymes and stop glycolysis. Together, the results of this study have uncovered a role for metabolism on MDSC suppression of CD4+ T cell responses. Thus, reestablishment of their metabolic activity may represent a mean to improve the functionality of CD4+ T cells during chronic *S. aureus* infection.

**Supplementary Information:**

The online version contains supplementary material available at 10.1007/s00018-023-04875-9.

## Introduction

*Staphylococcus aureus* is a major human pathogen and a leading cause of morbidity and mortality worldwide [1]. *S. aureus* can cause recurrent and chronic diseases such as chronic implant-related bone infections despite appropriate antibiotic treatment [2]. An important factor contributing to the chronicity and infection recurrence is the failure of the host to develop effective T cell responses against *S. aureus* [3, 4]. An understanding of the factors responsible for the host failure to generate effective cellular immunity against the pathogen is important for improving the management of chronic staphylococcal infections.

Several lines of evidence from animal models and human studies have converged to show that CD4+ T cells, in particular Th17 and Th1 subsets, are at the frontline of the adaptive immune response to *S. aureus* [5–10]. After recognition of antigen via the T cell receptor (TCR) and the receipt of costimulatory signals, CD4+ T cells become activated, undergo extensive proliferation and acquire effector functions, such as the ability to produce cytokines and other effector molecules [11]. Thus, although CD4+ T cells are not directly involved on bacterial killing, they can facilitate *S. aureus* clearance by increasing the recruitment of phagocytic cells to the site of infection and by enhancing their antimicrobial activity via the production of cytokines such as IFN-γ and IL-17 [8, 9, 12]. However, we have reported in previous studies that the functionality of CD4+ T cells become compromised with the progression of *S. aureus* infection toward chronicity [13]. CD4+ T cells dysfunction was manifested by a decreased production of effector cytokines and impaired proliferative responses upon TCR stimulation [13]. We have also reported that CD4+ T cell dysfunction observed during chronic *S. aureus* infection was attributed to extrinsic suppressive mechanisms exerted by MDSC [14, 15]. MDSC comprise an aberrant population of immature myeloid cells that accumulate during pathological conditions such as cancer and chronic infections and are potent inhibitors of T cell responses [16, 17]. MDSC have been reported to play an important role in chronic infections caused by *S. aureus* in humans as well as in experimental infection in mice [14, 18–20]. Therefore, targeting the immunosuppressive mechanisms exerted by MDSC could be a promising strategy to restore T cell dysfunction and facilitate *S. aureus* clearance during chronic infection. Although the molecular mechanisms underlying the suppressive effect of MDSC on T cell responses in the cancer setting have been addressed in many studies, the suppressive mechanisms of MDSC in chronic infections remained unclear. The aim of the current study was to elucidate the mechanisms through which MDSC mediate T cell dysfunction in the setting of *S. aureus* chronic infection*.*

Following antigen recognition, CD4+ T cells become activated and undergo metabolic reprograming, switching from an oxidative phosphorylation-dependent catabolic condition to a highly glycolytic state and adopt aerobic glycolysis in order to support the increased energetic demands required for cell proliferation and for the synthesis of effector molecules [21–25]. During aerobic glycolysis, glucose is converted to pyruvate that is reduced directly to lactate in the cytoplasm by the lactate dehydrogenase instead of entering the mitochondria for oxidation [25]. In this reaction, NAD+, which is an important co-factor for several glycolytic enzymes [26], is regenerated from NADH to enable the continuation of glycolysis [25]. Activated CD4+ T cells also need to constantly excrete the excess of lactate produced during aerobic glycolysis in order to avoid the reversal of the lactate dehydrogenase reaction that can shut down regeneration of NAD+ and discontinue the glycolytic process. Lactate is largely exported by activated CD4+ T cells via proton-linked monocarboxylate transports following a concentration gradient [27]. A limitation in the capacity of activated T cells to undergo metabolic shift toward aerobic glycolysis either by nutrient limitation or by inhibition of lactate excretion has been shown to impair T cell proliferation and cytokines production [21, 28–32].

We have recently shown that MDSC have an aberrant metabolism with very high glycolytic activity associated with the consumption of large amounts of glucose and the released of elevated levels of lactate in the extracellular milieu [15]. In the present study, we provide evidence that the lactate-rich local environment generated by MDSC in *S. aureus-*infected mice hinders CD4+ T cell activation by impairing NAD+ regeneration and disruption of glycolytic flux.

## Materials and methods

### Bacteria

The *S. aureus* strain 6850 was used in this study [33]. *S. aureus* was grown to the mid-log phase in brain heart infusion medium (BHI, Roth) at 37 °C with shaking (120 rpm), collected by centrifugation, washed with sterile PBS, and diluted to the required concentration.

### Experimental murine infection model and spleen cells isolation

Pathogen-free 10-week-old C57BL/6 female mice were purchased from Charles River (Germany) and maintained according to institutional guidelines in individually ventilated cages with food and water provided ad libitum. Mice were intravenously inoculated with 10^6^ CFU of *S. aureus* in 100 μl of PBS via a lateral tail vein, and sacrificed by CO_2_ asphyxiation at day 21 after bacterial inoculation. The spleen was removed from infected mice and single-cell suspensions were prepared by gently teasing the spleen tissue through a 100 µm pore size nylon cell strainer and the bone marrow was flushed out of both tibia and femur. Spleen and bone marrow cells were spun down, erythrocytes were lysed after incubation for 5 min at RT in ammonium-chloride-potassium lysing buffer (Lonza), washed with PBS + 10% FCS and resuspended in RPMI-1640 medium (Gibco) supplemented with 10% FCS and antibiotic–antimycotic (VWR International).

### Proliferation assay

Spleen cells were seeded in 96-well plates at a concentration of 5 × 10^6^/ml and incubated in the presence of 2 μg/ml of Armenian hamster anti-mouse CD3 plus 2 μg/ml of Syrian hamster anti-mouse CD28 antibodies (BD Pharmingen) for the specified time periods. Before stimulation, spleen cells were stained with CellTrace™ CFSE Cell Proliferation kit (Invitrogen) according to the manufacturer′s instruction. Cell proliferation was monitored by flow cytometry using CFSE dilution.

In some experiments, nicotinamide riboside (NR) (Sigma-Aldrich) at a concentration of 200 µM or the MCT1-selective inhibitor AZD3965 (Cayman) at a concentration of 100 nM was added to the cultures.

### Flow cytometry

Cell suspensions were incubated with anti-mouse CD16/32 (eBioscience) for 5 min at RT to block Fc receptors and stained for 20 min at 4 °C with anti-mouse CD4 antibodies (Biolegend). Cells were washed with PBS + 10% FCS and analyzed on a LSRII cytometer (Becton Dickinson).

For intracellular cytokines staining, cells were stained first with anti-mouse CD4 antibodies as described above, fixed for 15 min at RT with fixation buffer (Biolegend), washed twice with permeabilization buffer (BioLegend), and stained with anti-mouse IL-2 or anti-mouse IFN-γ antibodies. After washing with permeabilization buffer, cells were analyzed on a LSRII cytometer.

Cell viability was determined by flow cytometry using Zombie fixable viability kit according to the manufacturer's recommendations (BioLegend).

### MDSC depletion

Ly6C + Ly6G + MDSC were removed from the spleen cell suspensions prior to in vitro stimulation using the mouse Myeloid-Derived Suppressor Cell Isolation Kit (Miltenyi Biotec) according to the manufacturer's instructions. The negative fraction constituted the MDSC-depleted spleen cells population. Efficacy of depletion was > 90% as confirmed by flow cytometry.

### SCENITH assay

SCENITH was performed according to protocol published by Argüello et al*.* [34]. In brief, spleen cells isolated from either uninfected or *S. aureus-*infected mice at day 21 of infection were seeded in 96-well plates at a concentration of 5 × 10^6^/ml and incubated in the presence of 2 µg/ml of anti-CD3/anti plus CD28 antibodies for the specified times at 37 °C and 5% CO_2_. Cells incubated in medium without antibodies were used as control. DMSO or the metabolic inhibitors deoxy-d-glucose (2-DG, 100 mM) (Sigma-Aldrich), oligomycin (1 μM) (Sigma-Aldrich), or 2-DG plus oligomycin were added to the wells at the specified time points and further incubated for 1 h. Puromycin (Sigma-Aldrich) was added to the wells at a final concentration of 10 μg/ml during the last 30 min of incubation. After washing with cold PBS, cells were stained for surface CD4 as described above, fixed and permeabilized using the FOXP3 fixation and permeabilization kit (eBioScience) according to manufacturer’s instructions. Intracellular staining of puromycin was performed after incubation with anti-puromycin antibodies (Merk) for 1 h in permeabilization buffer. After washing with permeabilization buffer, cells were analyzed on a LSRII cytometer.

Glucose dependence was calculated as: 100 × (puromycin MFI levels in DMSO-treated cells − puromycin MFI levels in 2-DG-treated cells)/(puromycin MFI levels in CTR DMSO-treated cells − puromycin MFI levels in 2-DG + olygomycin-treated cells) and mitochondrial dependence was calculated as: 100 × (puromycin MFI levels in DMSO-treated cells − puromycin MFI levels in oligomycin-treated cells)/(puromycin MFI levels in CTR DMSO-treated cells − puromycin MFI levels in 2-DG + olygomycin-treated cells).

### Glut-1 staining

For intracellular staining of Glut-1, spleen cells were stained first with anti-mouse CD4 antibodies as described above, fixed for 15 min at RT with fixation buffer (Biolegend), washed twice with permeabilization buffer (BioLegend), and stained with anti-Glut-1 antibodies (Novus Biologicals). After washing with permeabilization buffer, cells were analyzed on a LSRII cytometer.

### Glucose uptake assay

Spleen cells were seeded in 96-well plates at a concentration of 5 × 10^6^/ml and incubated in the presence of 2 µg/ml of anti-CD3 plus anti-CD28 antibodies at 37 °C and 5% CO_2_. At the indicated time points, spleen cells were transferred to glucose-free RPMI-1640 medium supplemented with 300 μM 2-(N-[7-nitrobenz-2-oxa-1,3-diazol-4-yl] amino)-2-deoxyglucose (2-NBDG) (Thermo Fisher Scientific) and incubated for 30 min at 37 °C and 5% CO_2_. Cells were labelled for CD4 surface antigen, washed and analyzed by flow cytometry on a LSRII cytometer.

### Lactate measurement

Lactate concentrations were measured in culture supernatants or in homogenized spleen tissue using the commercially available Amplite Colorimetric l-Lactate Assay Kit (Elabscience) following the manufacture’s instruction.

### Isolation of CD4+ T cells

CD4+ T cells were isolated from cultured spleen cells using the mouse CD4+ T cell Isolation Kit (Miltenyi Biotec) according to the manufacturer's instructions.

### NAD/NADH ratio assay

NAD+ and NADH levels were measured in isolated CD4+ T cells using Amplite™ Colorimetric NAD/NADH Ration Assay kit according to the manufacturer’s instructions (AAT Bioquest).

### Analysis

Statistical analysis was performed using GraphPad Prism version 9.4.1 software. Differences between two groups were determined using a student *t* test. Groups of three or more were analyzed by one-way analysis of variance (ANOVA). Data were analyzed using FlowJo v9.3 software.

## Results

### Inhibition of CD4+ T cell responses by MDSC is linked to metabolism

**Fig. 1:**
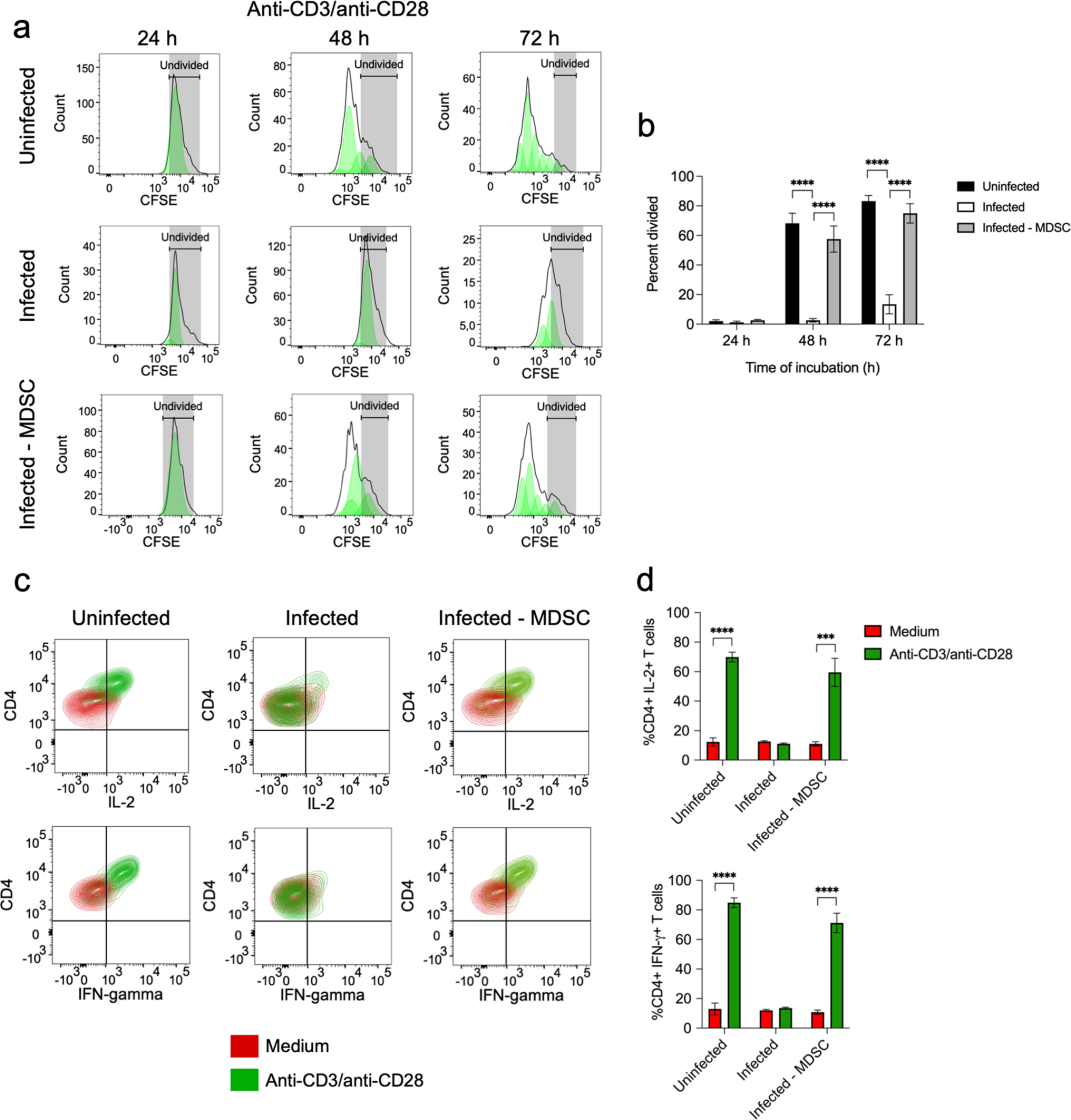
Suppression of CD4+ T cell responses by MDSC. **a** Flow cytometry histograms showing the kinetic of CD4+ T cells proliferation in spleen cells isolated from either uninfected (upper panels) or *S. aureus*-infected mice (middle panels) upon stimulation with anti-CD3/anti-CD28 antibodies. Proliferation of stimulated CD4+ T cells in the spleen cell population from *S. aureus*-infected mice that has been depleted of MDSC prior to stimulation is shown in the lower panels. The gating strategy is described in Supplementary Fig. S1. The percentage of divided CD4+ T cells in each group is shown in **b**. **c** Flow cytometry contour plots showing the intracellular staining of IL-2 (upper panels) and IFN-γ (lower panels) in CD4+ T cells within the spleen cell population isolated from uninfected (left panels) or *S. aureus*-infected (middle panels) mice as well as in MDSC-depleted spleen cells isolated from *S. aureus*-infected mice (lower panels) cultured for 72 h in the presence (green) or absence (red) of anti-CD3/anti-CD28 antibodies. The frequencies of CD4+ T cells expressing IL-2 (upper panel) or IFN-γ (lower panel) are shown in **d**. Each bar shows the mean ± SD of three independent experiments. ***p < 0.001, ****p < 0.0001

We have previously shown that the functionality of CD4+ T cells became compromised during chronic *S. aureus* infection [13]. Thus, whereas CD4+ T cells in spleen cells isolated from uninfected mice actively proliferated in response to stimulation with anti-CD3/anti-CD28 antibodies (Fig. 1a, b) and produced significant amounts of effector cytokines such as IL-2 and IFN-γ (Fig. 1c, d), CD4+ T cells in spleen cells isolated from *S. aureus-*infected mice were impaired in their capacity to proliferate (Fig. 1a, b) and to produce IL-2 and IFN-γ (Fig. 1c, d) upon stimulation with anti-CD3/anti-CD28 antibodies. We have also previously reported that immunosuppression of CD4+ T cell responses in *S. aureus*-infected mice was mediated by MDSC, an aberrant population of myeloid cells that expand during pathological conditions including chronic infections [14, 15]. MDSC accumulate in the spleen (Supplementary Fig. S2a and b) and bone marrow (Supplementary Fig. S2c, d) of *S. aureus-*infected mice and are responsible for T cell dysfunction [14, 15]. Indeed, CD4+ T cells in spleen cells isolated from infected mice recovered their capacity to proliferate (Fig. 1a, b) and to produce IL-2 and IFN-γ (Fig. 1c, d) in response to stimulation with anti-CD3/anti-CD28 antibodies after depletion of MDSC.

**Fig. 2:**
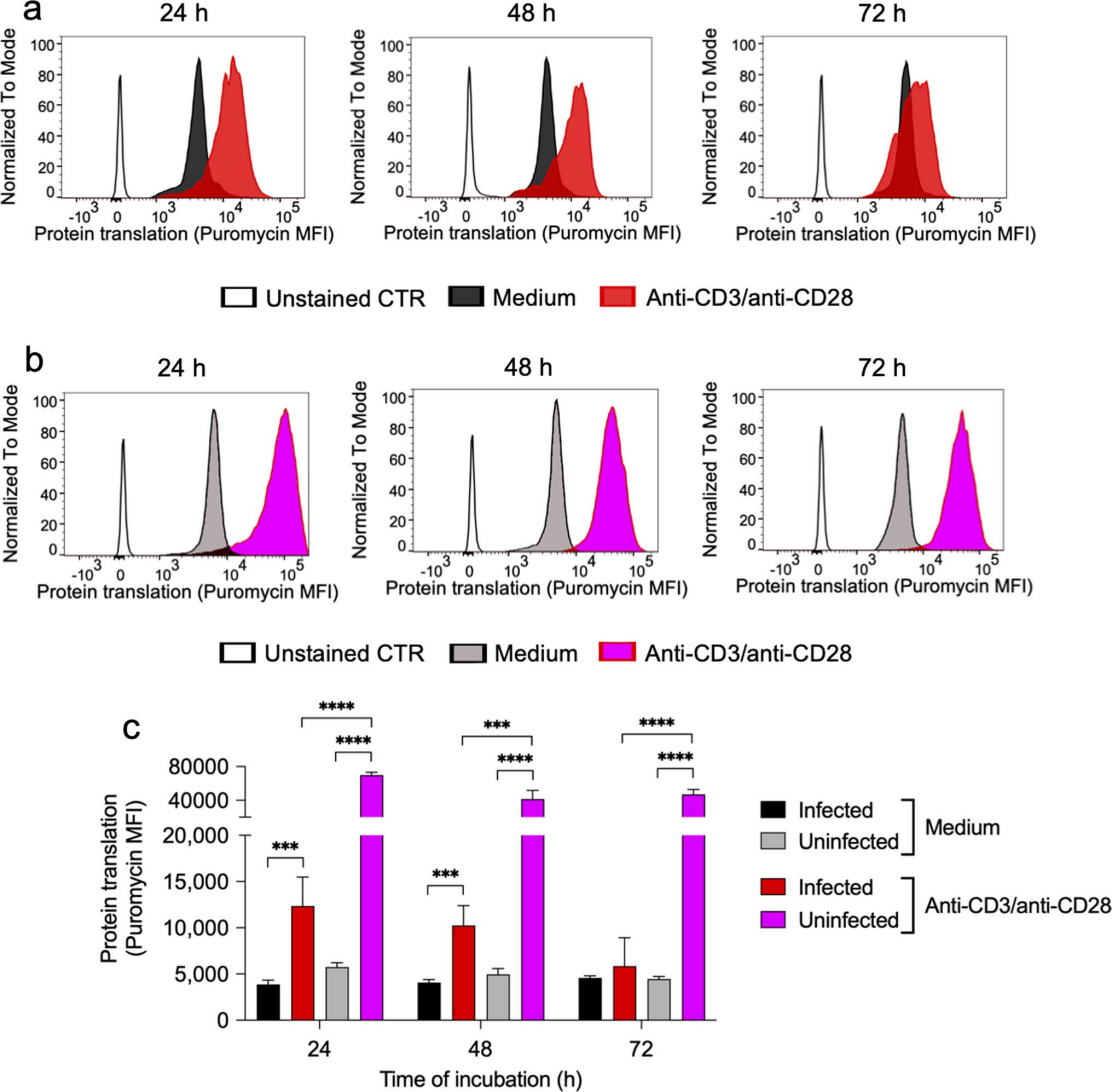
Metabolic activity of spleen CD4+ T cells from uninfected or from *S. aureus*-infected mice upon stimulation with anti-CD3/anti-CD28 antibodies determined by SCENITH. Flow cytometry histograms showing the levels of protein translation (puromycin MFI) in CD4+ T cells within spleen cells isolated from either *S. aureus*-infected (**a**) or from uninfected (**b**) mice in the presence or absence of anti-CD3/anti-CD28 antibodies. Unstained CD4+ T cells (without anti-puromycin antibodies) were used as control. The gating strategy is described in Supplementary Fig. S3. Quantification of protein translation levels (puromycin MFI) in the different groups is shown in **c**. Each bar shows the mean ± SD of five independent experiments. ***p < 0.001, ****p < 0.0001

In the study presented here, we investigated the mechanisms underlying the suppressive effect exerted by MDSC on CD4+ T cell responses in *S. aureus-*infected mice. Because activated CD4+ T cells undergo a shift in metabolism toward aerobic glycolysis which is absolutely required to synthesize intermediates required for cell proliferation and cytokine production [21–25], we considered the possibility that MDSC could impair CD4+ T cell responses in *S. aureus-*infected mice by limiting their capacity to undergo this metabolic shift. To investigate this hypothesis, we first assessed the metabolic activity of CD4+ T cells in spleen cells isolated from either *S. aureus-*infected or uninfected mice upon stimulation with anti-CD3/anti-CD28 antibodies using the recently described single-cell energetic metabolism by profiling translation inhibition (SCENITH) method [34]. SCENITH is based on the analysis of protein translation as surrogate of metabolic activity and enables to analyze the metabolic activity of specific cell subsets within heterogenous populations [34]. The degree of protein translation is determined by measuring the extent of puromycin incorporation into nascent polypeptide after staining with anti-puromycin antibodies using flow cytometry [34]. The results of this analysis showed that stimulation with anti-CD3/anti-CD28 antibodies induced a significant increase in the metabolic activity of CD4+ T cells in spleen cells isolated from *S. aureus-*infected mice (Fig. 2a, c), although to a significantly lower extent than that observed in CD4+ T cells in spleen cells isolated from uninfected animals (Fig. 2b, c). Furthermore, whereas the metabolic activity of anti-CD3/anti-CD28-stimulated CD4+ T cells in the spleen cells from uninfected mice was maintained at high levels during the whole incubation period, the metabolic activity progressively decreased in stimulated CD4+ T cells from infected mice during the incubation time (Fig. 2a, c).

### CD4+ T cells in the spleen of *S. aureus*-infected mice are not impaired in their capacity to up-regulate Glut-1 and to uptake glucose upon TCR stimulation

**Fig. 3:**
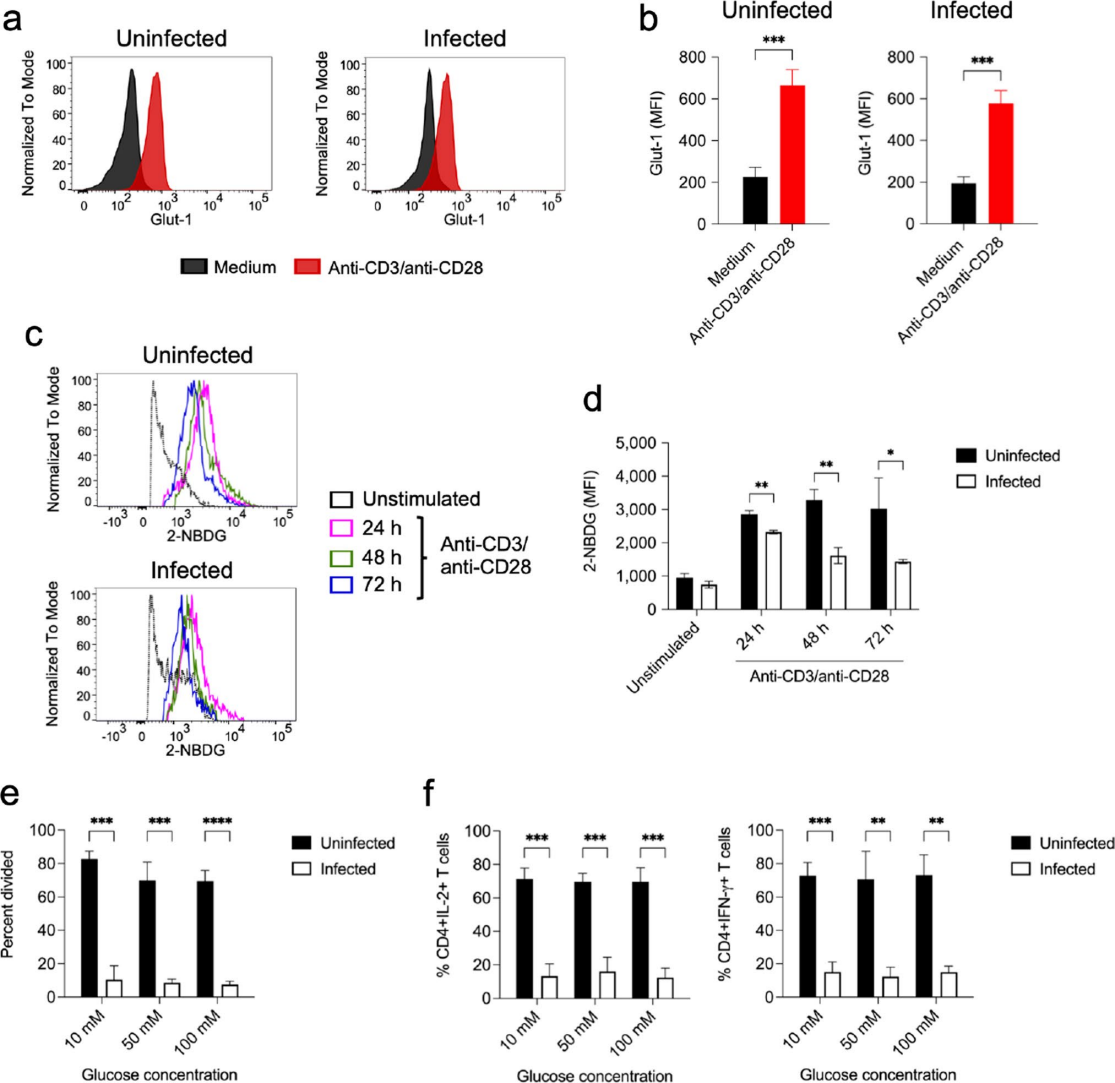
Expression of glucose transporter Glut-1 and glucose uptake by spleen CD4+ T cells from uninfected or from *S. aureus*-infected mice upon stimulation with anti-CD3/anti-CD28 antibodies. **a** Flow cytometry histograms showing the levels of intracellular Glut-1 expression in CD4+ T cells in spleen cells isolated from either uninfected (left panel) or *S. aureus*-infected (right panel) mice cultured for 24 h in the presence (red histogram) or absence (black histogram) of anti-CD3/anti-CD28 antibodies. Quantification of intracellular Glut-1 expression levels in the different groups is shown in **b**. **c** Flow cytometry histograms showing the kinetic of glucose uptake in CD4+ T cells in spleen cells isolated from either uninfected (upper panel) or *S. aureus*-infected (lower panel) mice unstimulated (black histograms) or stimulated for 24 h (pink histograms), 48 h (green histograms) or 72 h (blue histograms) with anti-CD3/anti-CD28 antibodies. Glucose uptake was determined using 2-NBDG. Quantification of glucose uptake in the different groups is shown in **d**. **e** percentage of divided CD4+ T cells in spleen cells from either uninfected (black bars) or *S. aureus*-infected (white bars) mice at 72 h upon stimulation with anti-CD3/anti-CD28 antibodies in the presence of either 10 mM, 50 mM or 100 mM glucose. **f** Frequencies of CD4+ T cells expressing IL-2 (left panel) or IFN-γ (right panel) within the spleen cell population isolated from uninfected (black bars) or *S. aureus*-infected (white bars) mice at 72 h upon stimulation with anti-CD3/anti-CD28 antibodies in the presence of either 10 mM, 50 mM or 100 mM glucose. Each bar shows the mean ± SD of three independent experiments. *p < 0.05, **p < 0.01, ***p < 0.001, ****p < 0.0001

The first step in glucose utilization by CD4+ T cells during metabolic reprograming upon activation is an increase in glucose uptake via up-regulation of the glucose transporter-1 (Glut-1) [35]. Therefore, we investigated if the lower metabolic activity of anti-CD3/anti-CD28-stimulated spleen CD4+ T cells from *S. aureus-*infected mice was due to an impaired capacity to up-regulate Glut-1. Determination of Glut-1 expression by flow cytometry analysis indicated that Glut-1 was significantly up-regulated in CD4+ T cells in spleen cells from infected mice at 24 h upon stimulation and to an extent similar to that observed in CD4+ T cells in the spleen from uninfected mice (Fig. 3a, b). We also measured the levels of glucose uptake by spleen CD4+ T cells from infected or uninfected mice in response to stimulation with anti-CD3/anti-CD28 antibodies using 2-NBDG. The results depicted in Fig. 3c, d show that although glucose uptake increased upon stimulation in CD4+ T cells from infected mice, the levels of glucose uptake were significantly lower than those of CD4+ T cells from uninfected animals. Since we have previously shown that MDSC present in the spleen of *S. aureus-*infected mice in high numbers consumed elevated amounts of glucose [15], we hypothesize that the lower amount of glucose taken up by stimulated CD4+ T cells from infected mice may result from a reduced glucose bioavailability due to the high consumption by the MDSC. To investigate this possibility, we determined the effect of adding increasing concentrations of glucose (ranging from 10 to 100 mM) in the culture medium on the proliferative response and production of IL-2 and IFN-γ by CD4+ T cells upon activation with anti-CD3/anti-CD28 antibodies. The results show that addition of increasing concentrations of extracellular glucose did not rescue the capacity of CD4+ T cells from infected mice to proliferate (Fig. 3e) or to produce cytokines (Fig. 3f) upon stimulation. Thus, glucose deprivation seems not to be the mechanism underlying the inhibitory effect exerted by MDSC on CD4+ T cell responses in the spleen of *S. aureus-*infected mice.

### CD4+ T cells in the spleen of *S. aureus*-infected mice are not able to sustain their glycolytic activity upon TCR stimulation

**Fig. 4:**
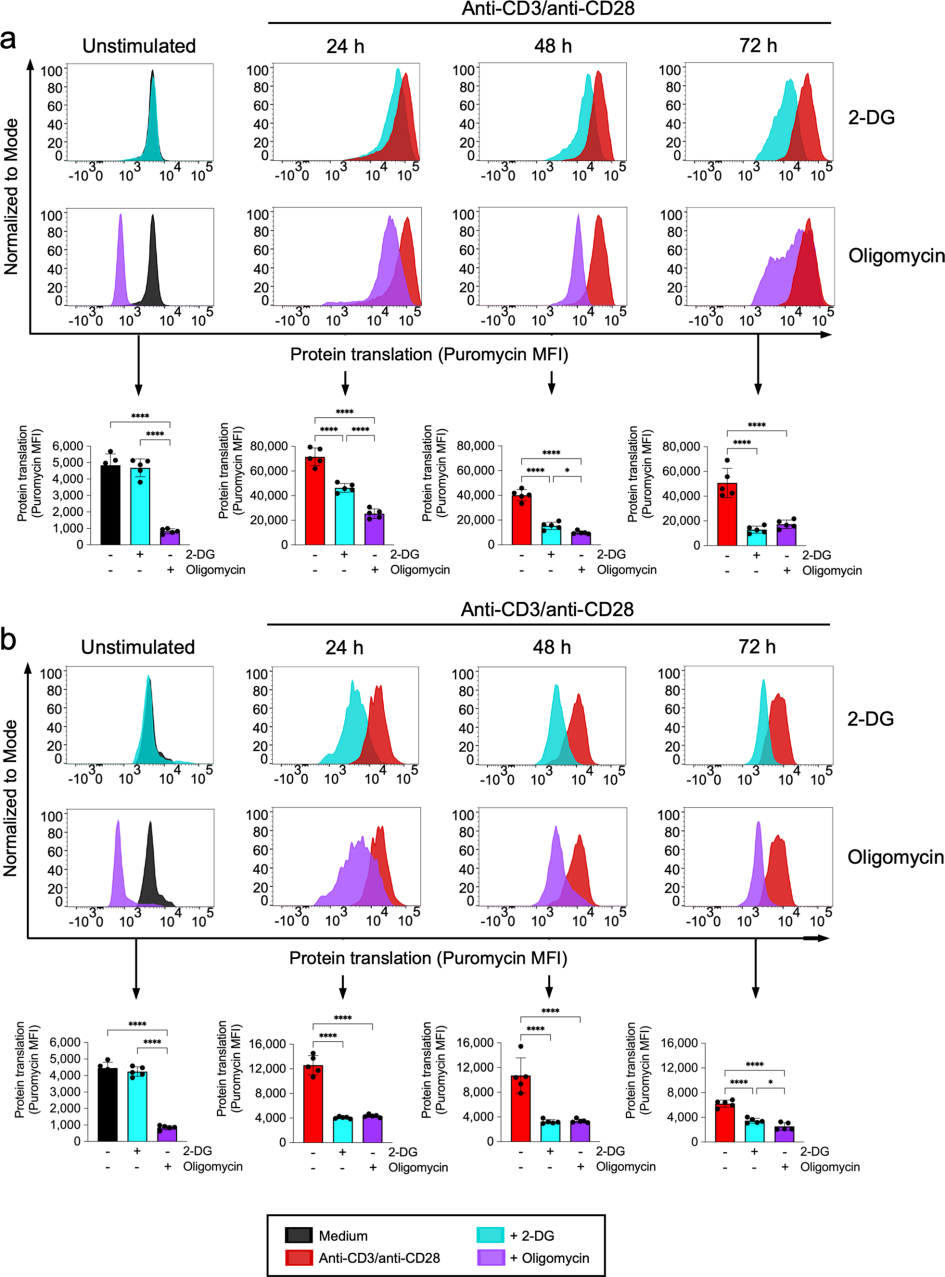
Metabolic profile of spleen CD4+ T cells from either uninfected or *S. aureus-*infected mice upon stimulation with anti-CD3/anti-CD28 antibodies. Flow cytometry histograms showing the levels of protein translation (puromycin MFI) in CD4+ T cells in spleen cells from uninfected (**a**) or *S. aureus-*infected (**b**) mice at the indicated times of stimulation with anti-CD3/anti-CD28 antibodies and treated with either the glycolysis inhibitor 2-DG (upper histograms) or the oxidative phosphorylation inhibitor oligomycin (lower histograms). Quantification of protein translation levels (puromycin MFI) in the different conditions is shown in the lower panels in **a** and **b**. *p < 0.05, ****p < 0.0001

**Fig. 5:**
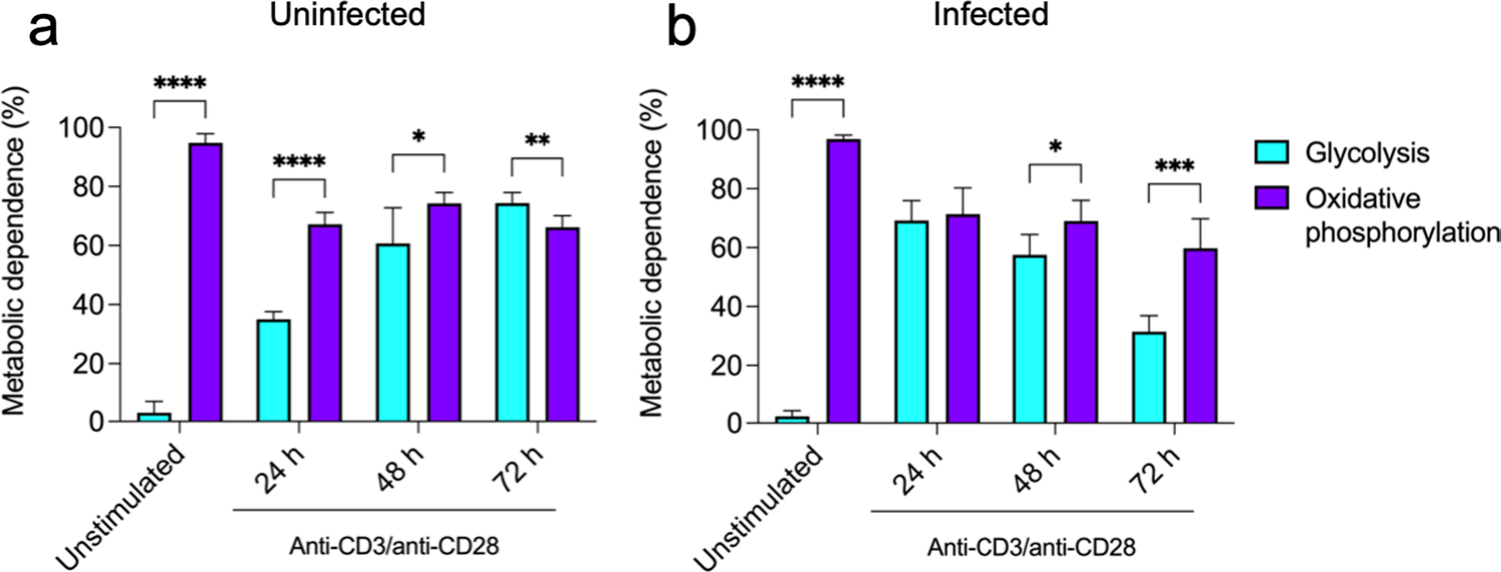
Contribution of glycolysis and oxidative phosphorylation to the metabolic activity of spleen CD4+ T cells from either uninfected or *S. aureus-*infected mice upon stimulation with anti-CD3/anti-CD28 antibodies. Metabolic dependence on glycolysis (cyan bars) or oxidative phosphorylation (purple bars) of spleen CD4+ T cells isolated from uninfected (**a**) or *S. aureus-*infected (**b**) mice upon stimulation with anti-CD3/anti-CD28 antibodies. Metabolic dependence was determined as described in the “Materials and methods” section. Each bar shows the mean ± SD of five independent experiments. *p < 0.05, **p < 0.01, ***p < 0.001, ****p < 0.0001

Because CD4+ T cells critically depend on reprogramming their metabolic activity toward aerobic glycolysis to meet the bioenergetic demands during activation [21–25], we next investigated whether CD4+ T cells in the spleen of *S. aureus-*infected mice were capable to reprogram their metabolic activity toward aerobic glycolysis upon stimulation with anti-CD3/anti-CD28 antibodies. To this end, we determined the effect of inhibiting glycolysis by treatment with 2-DG or oxidative phosphorylation by treatment with oligomycin on their metabolic activity using SCENITH. We also included in the analysis spleen cells from uninfected mice for comparison. In line with previously published data [36], we observed that the metabolic activity of unstimulated spleen CD4+ T cells from either uninfected (Figs. 4a, 5a) or *S. aureus*-infected (Figs. 4b, 5b) mice heavily relied on oxidative phosphorylation since inhibition of glycolysis by treatment with 2-DG did not have a significant effect on their metabolic activity but inhibition of oxidative phosphorylation using oligomycin resulted in marked reduction of their metabolic activity. However, the contribution of oxidative phosphorylation to the total metabolic activity was reduced upon anti-CD3/anti-CD28 antibodies stimulation (from > 80% in unstimulated to 50–60% in stimulated cells) in CD4+ T cells from uninfected mice whereas the contribution of glycolysis increased progressively during the stimulation time (Figs. 4a, 5a). In CD4+ T cells from infected mice, the contribution of oxidative phosphorylation to the total metabolic activity was also reduced upon anti-CD3/anti-CD28 antibodies stimulation (from > 80% in unstimulated to 50–60% in stimulated cells) (Figs. 4b, 5b). However, in contrast to the CD4+ T cells from uninfected mice, the contribution of glycolysis to the total metabolic activity increased sharply at 24 h and progressively decline during the incubation time (Figs. 4b, 5b). These data indicate that CD4+ T cells in the spleen of *S. aureus-*infected mice are capable to increase their glycolytic activity during the first 24 h upon TCR stimulation, but they are unable to sustain their glycolytic activity during the entire stimulation time.

### Inhibition of CD4+ T cell responses in the spleen cells of *S. aureus*-infected mice is mediated by increased extracellular lactate and reduction of intracellular NAD+/NADH ratio

**Fig. 6:**
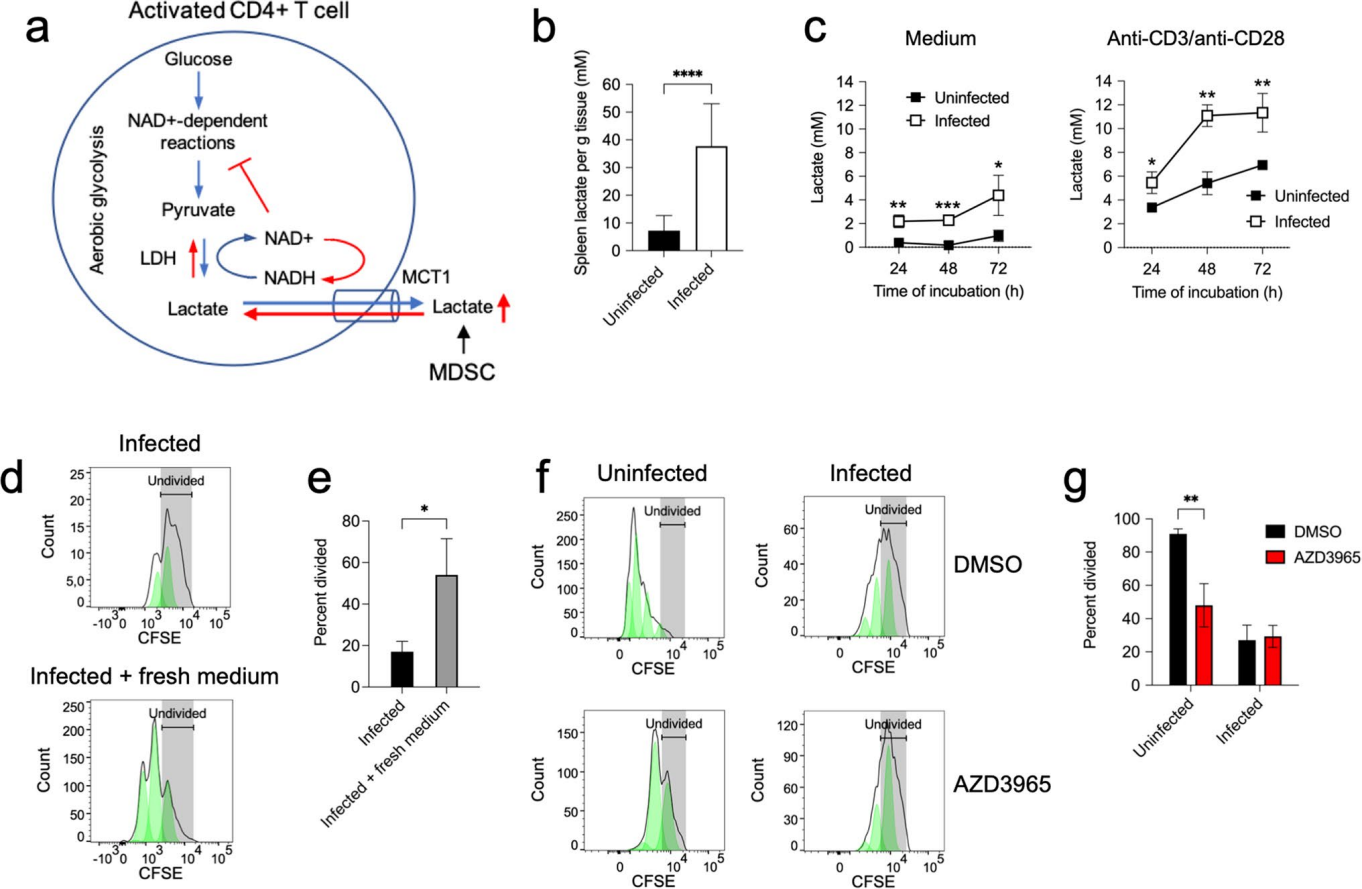
Lactate levels in spleen tissue and in the supernatant of cultured spleen cells from uninfected or *S. aureus-*infected mice **a** Scheme showing the potential role of lactate in the suppression of CD4+ T cell responses by MDSC in the spleen of *S. aureus-*infected mice. LDH (lactate dehydrogenase), NAD+ (oxidized nicotinamide adenine dinucleotide), NADH (reduced nicotinamide adenine dinucleotide), MCT1 (monocarboxylate transporter 1), MDSC (myeloid-derived suppressor cells). **b** Lactate concentration in spleen tissue of uninfected (black bar) or *S. aureus*-infected (white bar) mice. **c** Lactate concentration in the supernatant of spleen cells isolated from either uninfected (black symbols) or *S. aureus*-infected (white symbols) mice at progressing times of incubation in either medium alone (left panel) or with anti-CD3/anti-CD28 antibodies (right panel). **d** Flow cytometry histograms showing proliferation of CD4+ T cells in spleen cells isolated from *S. aureus*-infected mice at 72 h upon stimulation with anti-CD3/anti-CD28 antibodies without changing (upper panel) or after changing the medium every 24 h (lower panel). The percentage of divided CD4+ T cells in each group is shown in **e**. **f** Flow cytometry histograms showing proliferation of CD4+ T cell in spleen cells isolated either from uninfected (left histograms) or from *S. aureus*-infected (right histograms) mice at 72 h upon stimulation with anti-CD3/anti-CD28 antibodies in the presence of either the MCT1 inhibitor AZD3965 (100 nM) (lower histograms) or vehicle control DMSO (upper histograms). The percentage of divided CD4+ T cells in each group is shown in **g**. Each bar shows the mean ± SD of five independent experiments. *p < 0.05, **p < 0.005, ***p < 0.001, ****p < 0.0001

Several studies have reported that high levels of extracellular lactate can impair T cell proliferation and cytokines production [29, 37–39]. Lactate is a byproduct of aerobic glycolysis that is produced via lactate dehydrogenase by conversion of pyruvate and NADH into lactate and NAD+ [25]. NAD+ is an important cofactor that regulates glycolysis through its electron transfer function in redox reactions where is reversibly reduced to NADH [26]. NAD+ needs to be regenerated from NADH through the conversion of pyruvate to lactate by the lactate dehydrogenase to maintain active the glycolytic flux [25]. Because this reaction is reversible, lactate needs to be continuously excreted by activated T cells to enable the reaction to further move toward lactate and NAD+ production [25]. Lactate export is largely achieved by transport systems such as monocarboxylate transporter 1 (MCT1), which is a bidirectional proton-assisted transporter that cotransport protons and lactate anions through the plasma membrane depending on the concentration gradient [27, 40]. In lactate-rich environments, lactate accumulates within activated T cells leading to impaired NAD+ regeneration, blockage of glycolytic NAD^+^-dependent enzymatic reactions and drastic reduction of intermediates needed for proliferation [29]. Since we have previously shown that MDSC in the spleen of *S. aureus-*infected mice excreted high levels of lactate [15], we speculated that MDSC generate a lactate-rich environment in the spleen of infected mice that may hamper the lactate excretion and NAD+ regeneration of CD4+ T cells during activation. A graphic schema of this hypothesis is shown in Fig. 6a. To investigate this assumption, we first determined if the concentration of lactate differed between the spleen of *S. aureus-*infected and the spleen of uninfected mice. We found a significantly greater concentration of lactate in the spleen of infected mice compared to uninfected animals (Fig. 6b). Furthermore, spleen cells from infected mice produced significantly greater levels of lactate than those from uninfected animals after in vitro incubation in culture medium (Fig. 6c). The concentration of lactate increased in the culture supernatant of spleen cells from both infected and uninfected mice after stimulation with anti-CD3/anti-CD28 antibodies, although the lactate levels were significantly greater in spleen cells from infected than in those from uninfected mice (Fig. 6c). Removing the excess of lactate by changing the medium every 24 h resulted in significant recovery of proliferative capacity of CD4+ T cells in spleen from infected mice (Fig. 6d, e). On the other hand, abrogation of lactate excretion in stimulated spleen CD4+ T cells using the specific MCT1 inhibitor AZD3965 [41] resulted in significant reduced proliferation of CD4+ T cells from uninfected mice but did affect the unresponsiveness of CD4+ T cells from infected animals (Fig. 6f, g). These observations underscore the relevance of lactate excretion via MCT1 for a proper response of CD4+ T upon activation.

**Fig. 7:**
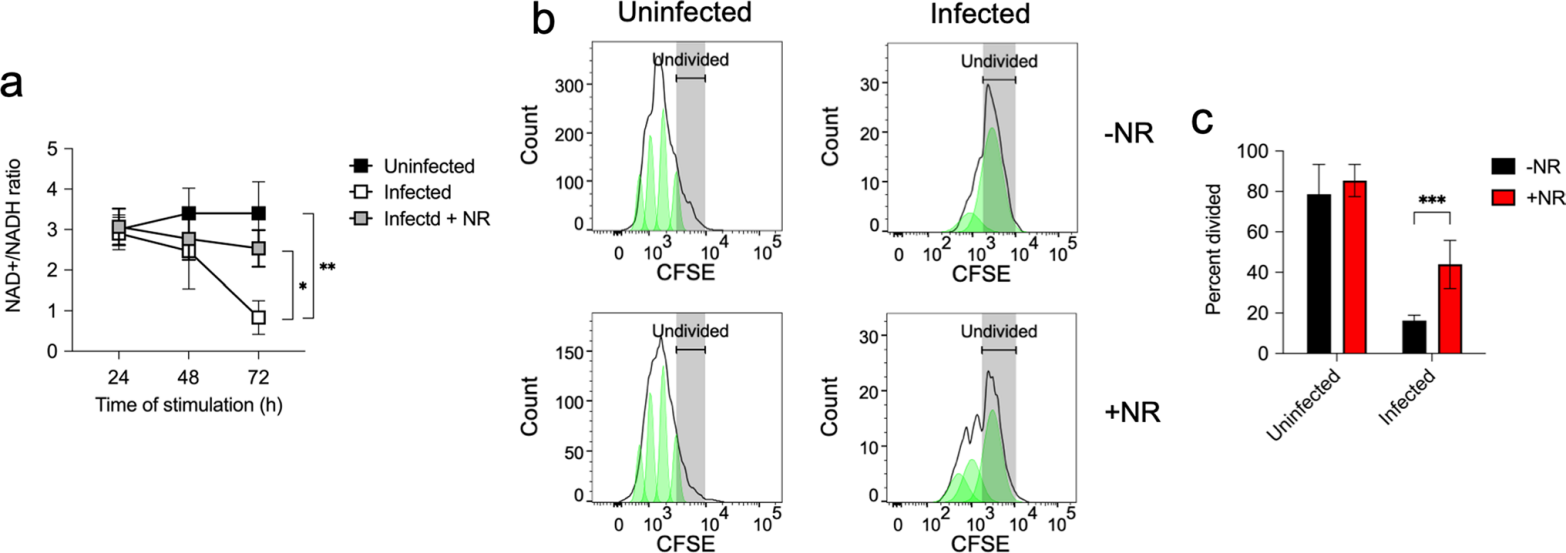
NAD+/NADH ratio in stimulated spleen CD4+ T cells from uninfected or *S. aureus*-infected mice. **a** NAD+/NADH ratio in CD4+ T cell isolated from cultured spleen cells from either uninfected (black symbols) or *S. aureus*-infected mice in the presence (white symbols) or absence (grey symbols) of 200 µM NR and stimulated with anti-CD3/anti-CD28 antibodies. Each symbol shows the mean ± SD of three independent experiments. **b** Flow cytometry histograms showing proliferation of CD4+ T cell in spleen cells isolated from either uninfected (left histograms) or *S. aureus*-infected (right histograms) mice at 72 h upon stimulation with anti-CD3/anti-CD28 antibodies in the absence (upper histogram) or presence (lower histograms) of NR (200 µM). The percentage of divided CD4+ T cells in each group is shown in **c**. Each bar shows the mean ± SD of five independent experiments. *p < 0.05, **p < 0.005, ***p < 0.001

Since high concentration of lactate can alter NAD+/NADH redox conditions in activated CD4+ T cells that can affect cell proliferation [29], we determined the intracellular NAD+/NADH ratio in stimulated CD4+ T cells in spleen cells isolated from either uninfected or *S. aureus-*infected mice. For this purpose, spleen cells were cultured in the presence or absence of anti-CD3/anti-CD28 antibodies and NAD+/NADH ratio was determined in isolated CD4+ T cells at 24 h, 48 h and 72 h of incubation. The results show that whereas the NAD+/NADH ratio was not significantly changed during the incubation period in stimulated CD4+ T cells from uninfected mice, NAD+/NADH ratio progressively declined with the time of incubation in stimulated CD4+ T cells from infected mice, indicating thus a redox shift from NAD+ to NADH (Fig. 7a). To further investigate the relevance of redox shift from NAD+ to NADH in the suppression of CD4+ T cells responses in the spleen of infected mice, we determined the effect of increasing NAD+ by supplementing the cell cultures with nicotinamide riboside (NR), a precursor that has been shown to increase the levels of NAD+ in cells [42]. We found that NR supplementation increased NAD+/NADH ratio in activated CD4+ T cells from infected mice (Fig. 7a). This increase was most evident at 72 h of incubation (Fig. 7a). We also observed that NR supplementation improved the capacity of spleen CD4+ T cells from infected mice to proliferate after stimulation with anti-CD3/anti-CD28 antibodies but has not major effect on the proliferative activity of CD4+ T cells from uninfected mice (Fig. 7b, c). Together, these results indicate that the high levels of lactate released by MDSC in the spleen of infected mice likely provoked a redox shift in activated CD4+ T cells that may be responsible, at least in part, for their unresponsiveness to TCR stimulation.

## Discussion

We have previously reported that MDSC expand during *S. aureus* infection and exert a suppressive effect of CD4+ T cells that support infection chronicity [14]. We have also shown that MDSC exhibited a dysregulated metabolism in chronically infected mice characterized by high glycolytic activity and release of large amounts of lactate [15]. In this study, we have investigated the mechanism underlying the immunosuppressive effect exerted by MDSC on CD4+ T cells responses during chronic *S. aureus* infection. Our results support the notion that the dysregulated metabolic activity of MDSC generates a lactate-rich local environment in the spleen of infected mice that is responsible for the suppression of CD4+ T cell responses. The sensitivity of CD4+ T cells toward high concentrations of exogenous lactate can be attributed to the specific metabolic reprograming that CD4+ T cells undergo upon activation to supply the energetic requirements associated with highly proliferative and biosynthetic processes [21, 23–25, 36, 43–45].

The primary metabolic adaptation of CD4+ T cells upon TCR stimulation is a switch from oxidative phosphorylation to aerobic glycolysis involving a marked increase in glucose uptake and a change in the fate of glucose carbons [23–25, 36, 44, 46–48]. In resting CD4+ T cells, glucose is converted into pyruvate that enters the TCA in the mitochondria to undergo oxidative phosphorylation leading to the production of ATP [49]. Activate CD4+ T cells, on the other hand, use predominantly aerobic glycolysis where a large proportion of pyruvate is not entering the TCA in the mitochondria but rather converted into lactate in the cell cytosol through the action of lactate dehydrogenase [49]. Although aerobic glycolysis is less efficient than oxidative phosphorylation yielding only four moles of ATP per glucose molecule, this pathway produces ATP faster than oxidative phosphorylation to meet the energy demand of rapidly dividing cells [22]. However, lactate molecules have to be exported by activated CD4+ T cells to ensure the continuation of glycolysis. Since lactate anions cannot cross the plasma membrane by free diffusion [50], lactate is exported via monocarboxylate transporter systems, which cotransport protons and lactate anions following a concentration gradient [27, 51]. A high concentration of extracellular lactate can reverse lactate flux and, in this way, interfere with CD4+ T cell activation. Indeed, inhibition of lactate export by monocarboxylate transporter MCT1 using pharmacological inhibitors have been shown to suppress T cell proliferation [52]. In this study, we provide evidence that the high concentration of extracellular lactate resulting from the metabolic activity of MDSC may be responsible for the suppression of CD4+ T cell responses in the spleen of *S. aureus-*infected mice. A situation similar to that described in the cancer setting where lactate released by tumor cells in the local environment opposes lactate efflux from T cells, leading to decreased cytokine production and cytotoxic activity which hampers the anti-tumor activity of effector T cells and favors tumor growth [31, 32, 53–55]. Lactate has been also implicated in dysregulation of immunometabolism during chronic inflammatory processes and autoimmune diseases [30, 39].

Regarding the mechanisms underlying the suppressive effect of extracellular lactate on CD4+ T cell responses, some studies have reported that lactic acid causes an acidification of the medium that can impair T cells activation [56–58]. For example, Calcinotto et al*.* [56] reported that acidic pH impaired cytolytic activity and cytokine secretion of T cells after TCR activation, although the mechanisms mediating these effects were not identified in that study. Other studies, however, have provided strong evidence for a pH-independent suppressive effect [29, 31]. Thus, treatment of T cells with hydrochloric acid resulted only in one half of the suppressive effect on T cell proliferation and cytokine production than that induced by lactic acid [31]. Furthermore, Quinn et al*.* [29] reported that lactate impairs T cell responses by inducing reductive stress, independently from extracellular acidification. They showed that, in lactate-rich conditions, export of lactate by activated T cells is blocked, leading to an accumulation of intracellular lactate that impedes recycling of NADH to NAD+ and the continuation of glycolysis [29]. NAD+ plays an important role in glycolysis, as it is required for enzymatic reactions such as glyceraldehyde 3-phosphate dehydrogenase and 3-phosphoglycerate dehydrogenase [59]. Therefore, a low NAD+/NADH ratio inhibits these reactions and dampen the glycolytic process. For this reason, to maintain active aerobic glycolysis, NAD+ needs to be continuously regenerated from NADH through the conversion of pyruvate to lactate by the lactate dehydrogenase [29]. Excretion of lactate is pivotal for this process since this reaction is reversible. The observation that lactate can impair T cell proliferation through a redox shift from NAD+ to NADH led us to question whether the detrimental effect of lactate on CD4+ T cell responses in the spleen of infected mice may be associated by redox shift form NAD+ to NADH resulting in altered NAD+/NADH ratio. We found that the NAD+/NADH ratio in activated CD4+ T cells was lower in spleen cells from *S. aureus-*infected mice than in spleen cells from uninfected animals. We also demonstrated that supplementing cultured spleen cells isolated form infected mice with the NAD+ precursor NR increased significantly the proliferative capacity of CD4+ T cell, further supporting a role for altered NAD+/NADH ratio on the suppression mechanism.

In summary, the results of our study suggest that release of high concentration of lactate by MDSC in the local microenvironment suppresses CD4+ T cell activation via blockade of lactate efflux, resulting in altered redox homeostasis and thereby disturbance of CD4+ T cell metabolism. Therefore, therapeutic manipulation of lactate levels or redox metabolism may open new approaches to overcome CD4+ T cells immunosuppression and improve immunity during chronic *S. aureus* infection. This could be accomplished for example by blocking the production of lactate using inhibitors of key enzymes involved in this process such as lactate dehydrogenase or by blocking lactate transport using inhibitors of the lactate transporters monocarboxylate transporters. These strategies have been shown to be effective at reducing lactate levels in the tumor environment in preclinical studies [60]. However, these therapeutic strategies still face many challenges and may have unintended adverse consequences due to their off-target effects and the important role of lactate in the maintenance of cellular functions and immune regulation. Alternatively, boosting NAD+ content either by supplementation of NAD+ precursors such as nicotinamide mononucleotide may provide another strategy to ameliorate T cells immunosuppression [61]. However, further studies are needed to determine the optimal dosing and effects of NAD+ supplementation on immune function during infection.

One major limitation of this study is that the experiments have been performed with ex vivo isolated spleen cells, which may not accurately reflect the complex interactions and responses that occur in the in vivo system. Furthermore, although MDSCs have been extensively studied in mouse models, the role of MDSC in humans is less well-defined. Human and mouse MDSC differ in the phenotypic markers that characterize the specific MDSC subsets as well as in some physiological aspects [62]. However, they also exhibit some similarities for example in the expression of cell surface markers such as CD11b and in their immunosuppressive functions on T cell activation [62]. Overall, while the role of MDSCs in human diseases is still being elucidated, there is growing evidence that these cells play an important role in immune regulation and disease progression. Further research is needed to fully understand the function of human MDSCs and develop effective therapies that target these cells.

## Supplementary Information

Below is the link to the electronic supplementary material.

Supplementary file1 (PDF 1054 KB)

## Data Availability

All data generated or analyzed during this study are included in this article and its supplementary information file. Additionally, data are available from the corresponding author upon request.

## References

[1] Tong SY, Davis JS, Eichenberger E, Holland TL, Fowler VG (2015). *Staphylococcus aureus* infections: epidemiology, pathophysiology, clinical manifestations, and management. Clin Microbiol Rev.

[2] Peel TN, Cheng AC, Buising KL, Choong PF (2012). Microbiological aetiology, epidemiology, and clinical profile of prosthetic joint infections: are current antibiotic prophylaxis guidelines effective?. Antimicrob Agents Chemother.

[3] Deng J, Zhang BZ, Chu H, Wang XL, Wang Y, Gong HR (2021). Adenosine synthase A contributes to recurrent *Staphylococcus aureus* infection by dampening protective immunity. EBioMedicine.

[4] Sanchez M, Kolar SL, Muller S, Reyes CN, Wolf AJ, Ogawa C (2017). O-Acetylation of peptidoglycan limits helper T cell priming and permits *Staphylococcus aureus* reinfection. Cell Host Microbe.

[5] Broker BM, Mrochen D, Peton V (2016). The T cell response to *Staphylococcus aureus*. Pathogens.

[6] Karauzum H, Datta SK (2017). Adaptive immunity against *Staphylococcus aureus*. Curr Top Microbiol Immunol.

[7] Utay NS, Roque A, Timmer JK, Morcock DR, DeLeage C, Somasunderam A (2016). MRSA infections in HIV-infected people are associated with decreased MRSA-specific Th1 immunity. PLoS Pathog.

[8] Brown AF, Murphy AG, Lalor SJ, Leech JM, O'Keeffe KM, Mac Aogain M (2015). Memory Th1 cells are protective in invasive *Staphylococcus aureus* infection. PLoS Pathog.

[9] Lin L, Ibrahim AS, Xu X, Farber JM, Avanesian V, Baquir B (2009). Th1-Th17 cells mediate protective adaptive immunity against *Staphylococcus aureus* and *Candida albicans* infection in mice. PLoS Pathog.

[10] Kolata JB, Kuhbandner I, Link C, Normann N, Vu CH, Steil L (2015). The fall of a dogma? Unexpected high T-cell memory response to *Staphylococcus aureus* in humans. J Infect Dis.

[11] Zhu J, Yamane H, Paul WE (2010). Differentiation of effector CD4 T cell populations (*). Annu Rev Immunol.

[12] Montgomery CP, Daniels M, Zhao F, Alegre ML, Chong AS, Daum RS (2014). Protective immunity against recurrent *Staphylococcus aureus* skin infection requires antibody and interleukin-17A. Infect Immun.

[13] Ziegler C, Goldmann O, Hobeika E, Geffers R, Peters G, Medina E (2011). The dynamics of T cells during persistent *Staphylococcus aureus* infection: from antigen-reactivity to in vivo anergy. EMBO Mol Med.

[14] Tebartz C, Horst SA, Sparwasser T, Huehn J, Beineke A, Peters G (2015). A major role for myeloid-derived suppressor cells and a minor role for regulatory T cells in immunosuppression during *Staphylococcus aureus* infection. J Immunol.

[15] Dietrich O, Heinz A, Goldmann O, Geffers R, Beineke A, Hiller K (2022). Dysregulated immunometabolism is associated with the generation of myeloid-derived suppressor cells in *Staphylococcus aureus* chronic infection. J Innate Immun.

[16] Veglia F, Sanseviero E, Gabrilovich DI (2021). Myeloid-derived suppressor cells in the era of increasing myeloid cell diversity. Nat Rev Immunol.

[17] Medina E, Hartl D (2018). Myeloid-derived suppressor cells in infection: a general overview. J Innate Immun.

[18] Heim CE, Vidlak D, Scherr TD, Kozel JA, Holzapfel M, Muirhead DE (2014). Myeloid-derived suppressor cells contribute to *Staphylococcus aureus* orthopedic biofilm infection. J Immunol.

[19] Heim CE, Vidlak D, Scherr TD, Hartman CW, Garvin KL, Kielian T (2015). IL-12 promotes myeloid-derived suppressor cell recruitment and bacterial persistence during *Staphylococcus aureus* orthopedic implant infection. J Immunol.

[20] Heim CE, Vidlak D, Odvody J, Hartman CW, Garvin KL, Kielian T (2018). Human prosthetic joint infections are associated with myeloid-derived suppressor cells (MDSCs): implications for infection persistence. J Orthop Res.

[21] Wang R, Green DR (2012). Metabolic reprogramming and metabolic dependency in T cells. Immunol Rev.

[22] Vander Heiden MG, Cantley LC, Thompson CB (2009). Understanding the Warburg effect: the metabolic requirements of cell proliferation. Science.

[23] van der Windt GJ, Pearce EL (2012). Metabolic switching and fuel choice during T-cell differentiation and memory development. Immunol Rev.

[24] Palmer CS, Ostrowski M, Balderson B, Christian N, Crowe SM (2015). Glucose metabolism regulates T cell activation, differentiation, and functions. Front Immunol.

[25] Lunt SY, Vander Heiden MG (2011). Aerobic glycolysis: meeting the metabolic requirements of cell proliferation. Annu Rev Cell Dev Biol.

[26] Navas LE, Carnero A (2021). NAD(+) metabolism, stemness, the immune response, and cancer. Signal Transduct Target Ther.

[27] Halestrap AP, Wilson MC (2012). The monocarboxylate transporter family—role and regulation. IUBMB Life.

[28] Wei J, Raynor J, Nguyen TL, Chi H (2017). Nutrient and metabolic sensing in T cell responses. Front Immunol.

[29] Quinn WJ, Jiao J, TeSlaa T, Stadanlick J, Wang Z, Wang L (2020). Lactate limits T cell proliferation via the NAD(H) redox state. Cell Rep.

[30] Pucino V, Bombardieri M, Pitzalis C, Mauro C (2017). Lactate at the crossroads of metabolism, inflammation, and autoimmunity. Eur J Immunol.

[31] Fischer K, Hoffmann P, Voelkl S, Meidenbauer N, Ammer J, Edinger M (2007). Inhibitory effect of tumor cell-derived lactic acid on human T cells. Blood.

[32] Brand A, Singer K, Koehl GE, Kolitzus M, Schoenhammer G, Thiel A (2016). LDHA-associated lactic acid production blunts tumor immunosurveillance by T and NK cells. Cell Metab.

[33] Fraunholz M, Bernhardt J, Schuldes J, Daniel R, Hecker M, Sinha B (2013). Complete genome sequence of *Staphylococcus aureus* 6850, a highly cytotoxic and clinically virulent methicillin-sensitive strain with distant relatedness to prototype strains. Genome Announc.

[34] Arguello RJ, Combes AJ, Char R, Gigan JP, Baaziz AI, Bousiquot E (2020). SCENITH: a flow cytometry-based method to functionally profile energy metabolism with single-cell resolution. Cell Metab.

[35] Macintyre AN, Gerriets VA, Nichols AG, Michalek RD, Rudolph MC, Deoliveira D (2014). The glucose transporter Glut1 is selectively essential for CD4 T cell activation and effector function. Cell Metab.

[36] Pearce EL, Pearce EJ (2013). Metabolic pathways in immune cell activation and quiescence. Immunity.

[37] Angelin A, Gil-de-Gomez L, Dahiya S, Jiao J, Guo L, Levine MH (2017). Foxp3 reprograms T cell metabolism to function in low-glucose, high-lactate environments. Cell Metab.

[38] Caslin HL, Abebayehu D, Pinette JA, Ryan JJ (2021). Lactate is a metabolic mediator that shapes immune cell fate and function. Front Physiol.

[39] Pucino V, Certo M, Bulusu V, Cucchi D, Goldmann K, Pontarini E (2019). Lactate buildup at the site of chronic inflammation promotes disease by inducing CD4(+) T cell metabolic rewiring. Cell Metab.

[40] Haas R, Smith J, Rocher-Ros V, Nadkarni S, Montero-Melendez T, D'Acquisto F (2015). Lactate regulates metabolic and pro-inflammatory circuits in control of T cell migration and effector functions. PLoS Biol.

[41] Bola BM, Chadwick AL, Michopoulos F, Blount KG, Telfer BA, Williams KJ (2014). Inhibition of monocarboxylate transporter-1 (MCT1) by AZD3965 enhances radiosensitivity by reducing lactate transport. Mol Cancer Ther.

[42] Mehmel M, Jovanovic N, Spitz U (2020). Nicotinamide riboside—the current state of research and therapeutic uses. Nutrients.

[43] Smith-Garvin JE, Koretzky GA, Jordan MS (2009). T cell activation. Annu Rev Immunol.

[44] Pearce EL, Poffenberger MC, Chang CH, Jones RG (2013). Fueling immunity: insights into metabolism and lymphocyte function. Science.

[45] Maciolek JA, Pasternak JA, Wilson HL (2014). Metabolism of activated T lymphocytes. Curr Opin Immunol.

[46] Fox CJ, Hammerman PS, Thompson CB (2005). Fuel feeds function: energy metabolism and the T-cell response. Nat Rev Immunol.

[47] Almeida L, Lochner M, Berod L, Sparwasser T (2016). Metabolic pathways in T cell activation and lineage differentiation. Semin Immunol.

[48] Menk AV, Scharping NE, Moreci RS, Zeng X, Guy C, Salvatore S (2018). Early TCR signaling induces rapid aerobic glycolysis enabling distinct acute T cell effector functions. Cell Rep.

[49] Chapman NM, Boothby MR, Chi H (2020). Metabolic coordination of T cell quiescence and activation. Nat Rev Immunol.

[50] Poole RC, Halestrap AP (1993). Transport of lactate and other monocarboxylates across mammalian plasma membranes. Am J Physiol.

[51] Halestrap AP, Price NT (1999). The proton-linked monocarboxylate transporter (MCT) family: structure, function and regulation. Biochem J.

[52] Murray CM, Hutchinson R, Bantick JR, Belfield GP, Benjamin AD, Brazma D (2005). Monocarboxylate transporter MCT1 is a target for immunosuppression. Nat Chem Biol.

[53] Ippolito L, Morandi A, Giannoni E, Chiarugi P (2019). Lactate: a metabolic driver in the tumour landscape. Trends Biochem Sci.

[54] Certo M, Tsai CH, Pucino V, Ho PC, Mauro C (2021). Lactate modulation of immune responses in inflammatory versus tumour microenvironments. Nat Rev Immunol.

[55] Watson MJ, Delgoffe GM (2022). Fighting in a wasteland: deleterious metabolites and antitumor immunity. J Clin Invest.

[56] Calcinotto A, Filipazzi P, Grioni M, Iero M, De Milito A, Ricupito A (2012). Modulation of microenvironment acidity reverses anergy in human and murine tumor-infiltrating T lymphocytes. Cancer Res.

[57] Lardner A (2001). The effects of extracellular pH on immune function. J Leukoc Biol.

[58] Pilon-Thomas S, Kodumudi KN, El-Kenawi AE, Russell S, Weber AM, Luddy K (2016). Neutralization of tumor acidity improves antitumor responses to immunotherapy. Cancer Res.

[59] Verdin E (2015). NAD(+) in aging, metabolism, and neurodegeneration. Science.

[60] Wang ZH, Peng WB, Zhang P, Yang XP, Zhou Q (2021). Lactate in the tumour microenvironment: from immune modulation to therapy. EBioMedicine.

[61] Wang Y, Wang F, Wang L, Qiu S, Yao Y, Yan C (2021). NAD(+) supplement potentiates tumor-killing function by rescuing defective TUB-mediated NAMPT transcription in tumor-infiltrated T cells. Cell Rep.

[62] Vanhaver C, van der Bruggen P, Bruger AM (2021). MDSC in mice and men: mechanisms of immunosuppression in cancer. J Clin Med.

