# Supplementary material for: Myeloid-derived suppressor cells impair CD4+ T cell responses during chronic Staphylococcus aureus infection via lactate metabolism
Source: Cell Mol Life Sci. 2023 Jul 22;80(8):221. doi: 10.1007/s00018-023-04875-9 (PMC10363054; doi:10.1007/s00018-023-04875-9)
Supplement: Supplementary file 1 — Supplementary file1 (PDF 1054 KB) [file 18_2023_4875_MOESM1_ESM.pdf]

Supplementary Figure S1

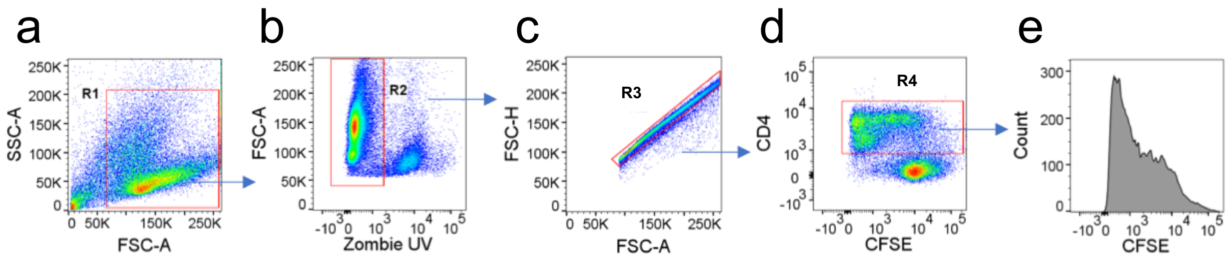

Fig. S1. Gating strategies for analysis of proliferating CD4+ T cells. **a** Forward scatter (FSC-A) and side scatter (SSC-A) was used to exclude cell debris (region R1). **b** Exclusion of dead cells using viability dye (region R2). **c** Doublet exclusion gate based upon FSC-H vs FSC-A (region R3). **d** R4 gate was drawn around the CD4+ T cell population. **e** Histogram showing the CFSE fluorescence intensity (FL1) within the CD4+ T cell population.

Supplementary Figure S2

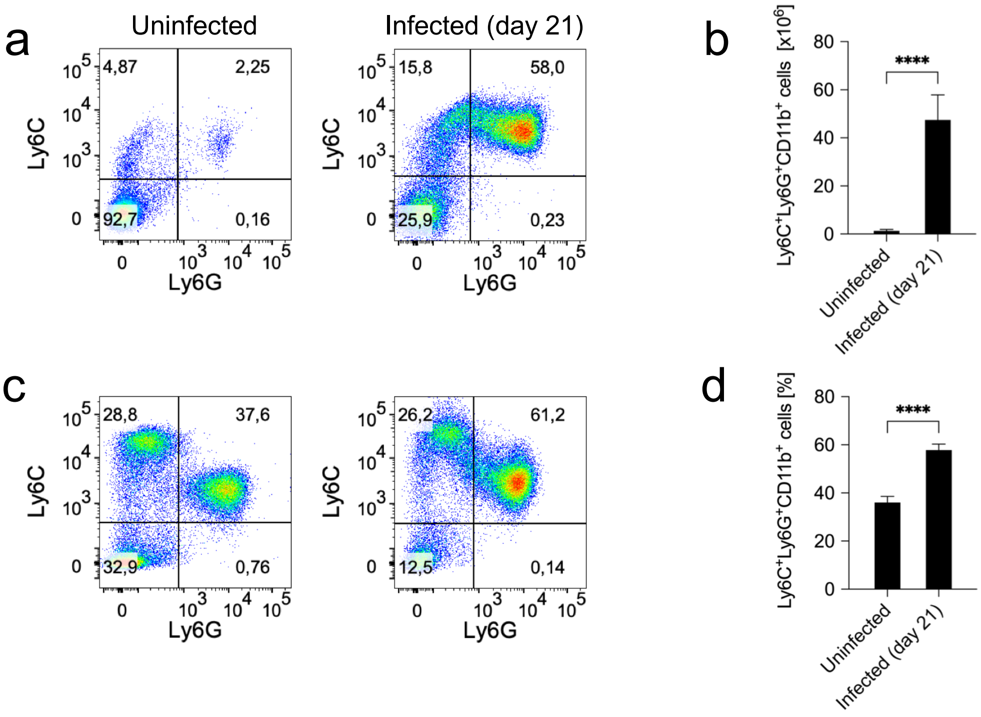

Fig. S2. Expansion of MDSC in the spleen and bone marrow of *S. aureus*-infected mice. **a** Flow cytometry analysis showing the frequency of Ly6C+Ly6G+CD11b+ MDSC in the spleen of uninfected (left panel) or *S. aureus*-infected (right panel) mice at day 21 of infection. **b** Absolute numbers of Ly6C+Ly6G+CD11b+ MDSC in the spleen of uninfected or *S. aureus*-infected mice at day 21 of infection (*n*=5). **c** Flow cytometry analysis showing the frequency of Ly6C+Ly6G+CD11b+ MDSC in the bone marrow of uninfected (left panel) or *S. aureus*-infected (right panel) mice at day 21 of infection. **d** Frequency of Ly6C+Ly6G+CD11b+ MDSC in bone marrow of uninfected or *S. aureus*-infected mice at day 21 of infection (*n*=5). ****, $p < 0.0001$.

Supplementary Figure S3

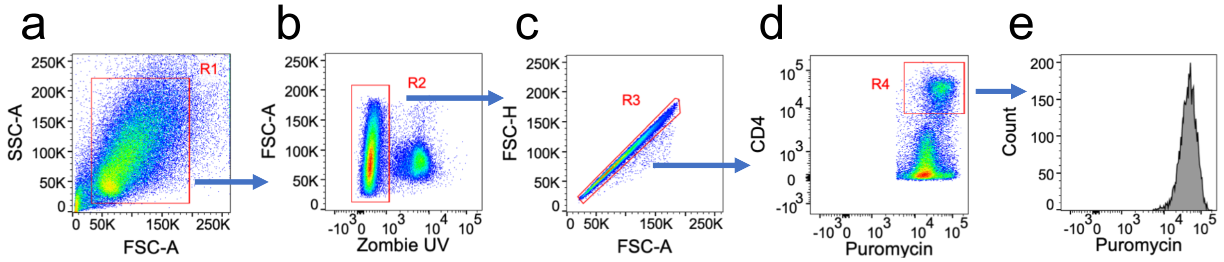

Fig. S3. Gating strategies for SCENITH analysis. **a** forward scatter (FSC-A) and side scatter (SSC-A) was used to exclude cell debris (region R1). **b** Exclusion of dead cells using viability dye (region R2). **c** Doublet exclusion gate based upon FSC-H vs FSC-A (region R3). **d** R4 gate was drawn around the CD4+ cells population. **e** Histogram showing the intensity anti-puromycin staining within the CD4+ cell population.
